# Real-world retrospective study on the efficacy and safety of anti-IgE therapy combined with rush immunotherapy in allergic asthma

**DOI:** 10.3389/falgy.2025.1717446

**Published:** 2025-12-15

**Authors:** Wenchao Zhang, Dan Liu, Wenjin Du, Zhaoji Meng, Xianghua Lin, Weili Guo, Yuanxi Jin, Siqin Wang, Qiuxing Zhang

**Affiliations:** 1 Department of Allergy, Henan Provincial People’s Hospital, Zhengzhou University, Zhengzhou, China; 2 Department of Respiratory and Critical Care Medicine, Henan Provincial People’s Hospital, Zhengzhou University, Zhengzhou, China

**Keywords:** rush immunotherapy, omalizumab, mite allergen, allergic asthma, safety

## Abstract

**Background:**

Research on the combination of biologics with rush immunotherapy (RIT) remains scarce, particularly regarding safety and efficacy data in pediatric and hypersensitive populations undergoing rapid desensitization or concurrent biologic therapy. Furthermore, regarding RIT, it remains unclear which patients can effectively reduce the occurrence of adverse reactions when combined with biologics, and which patients fail to achieve such a reduction with this combination therapy.

**Methods:**

This retrospective study analyzed 202 patients with mite-induced allergic asthma (2018–2024) receiving RIT alone (*n* = 133) or omalizumab-pretreated RIT (RIT + Omb-pre, *n* = 69). Stratified analyses were conducted based on age, mite sIgE levels, total IgE(T-IgE) levels, and sIgE to T-IgE ratios. Outcomes included systemic adverse reaction (SR) rates, RIT completion rates, improvements in clinical parameters following omalizumab intervention, and 1-year follow-up efficacy across subgroups.

**Results:**

Both regimens were well tolerated, with no grade ≥3 SRs observed. Compared to RIT alone, RIT + Omb-pre significantly reduced SR incidence (*p* < 0.05) and showed a trend toward higher target peak concentration completion rates (*p* = 0.054). Age-stratified analysis revealed higher SR risks in children/teenager patients vs. adults. Subgroup analyses further demonstrated that SR incidence correlated positively with mite sIgE levels and sIgE/T-IgE ratio (*p* < 0.05), but not with T-IgE. Patients with low-risk biomarkers (sIgE grades 1–2 and sIgE/T-IgE <10%) exhibited minimal SR incidence unaffected by omalizumab, whereas high-risk subgroups (sIgE grades 3–6 and sIgE/T-IgE ≥10%) showed significantly elevated SR incidence, which was markedly mitigated by omalizumab (*p* < 0.05).Subgroup with sIgE/T-IgE ratios >16% achieved substantially greater improvements in ACQ scores and daily medication burden compared to those with ratios <16% during the 12-month intervention. Furthermore, this study reaffirmed the age-dependent efficacy correlation, with pediatric patients demonstrating superior therapeutic outcomes to adult patients.

**Conclusions:**

Regarding the safety of dust mite rush immunotherapy for allergic asthma, Omalizumab significantly reduces the incidence of SRs in high-risk populations (sIgE grades 3–6 and sIgE/T-IgE ≥10%), whereas it demonstrates limited efficacy in low-risk subgroups (sIgE grades 1–2 and sIgE/T-IgE <10%).

## Introduction

Allergen-specific immunotherapy (AIT) has evolved over a century. With the maturation of allergen extract preparation technology and innovations in desensitization protocols, an increasing number of global authoritative health organizations have recognized the critical role of AIT in managing allergic diseases (1–5). These organizations recommend initiating desensitization therapy at an early stage for conditions such as allergic rhinitis and asthma to prevent irreversible tissue damage caused by recurrent disease exacerbations. However, the adoption of AIT in clinical practice within China remains suboptimal. Many patients struggle to adhere to conventional protocols due to drawbacks such as prolonged treatment duration, delayed efficacy, and frequent injections, leading to high discontinuation rates (6).

While conventional desensitization protocols requires approximately four months of weekly injections to complete the dose escalation phase, rush immunotherapy (RIT) accomplishes this process within a single week. This accelerated approach significantly reduces the number of injections, shortens the time to onset of therapeutic effect, and improves patient compliance (7–9). However, while this “aggressive” therapeutic approach offers clinical benefits, it inevitably increases the risk of allergen administration-related adverse reactions, limiting its widespread clinical adoption (8). In recent years, the clinical application of biologics such as omalizumab and dupilumab has demonstrated significant potential when combined with desensitization therapy (7, 10–12). Nevertheless, research on the combination of biologics with RIT remains scarce, particularly regarding safety and efficacy data in pediatric and hypersensitive populations undergoing rapid desensitization or concurrent biologic therapy. Furthermore, regarding RIT, it remains unclear which patients can effectively reduce the occurrence of adverse reactions when combined with biologics, and which patients fail to achieve such a reduction with this combination therapy. In this retrospective real-world study, we compared the efficacy and safety of RIT combined with omalizumab pretreatment vs. RIT alone in mite-induced asthma patients (both hypersensitive and pediatric populations) treated at our center during recent years.

## Materials and methods

### Patients

**Figure 1:**
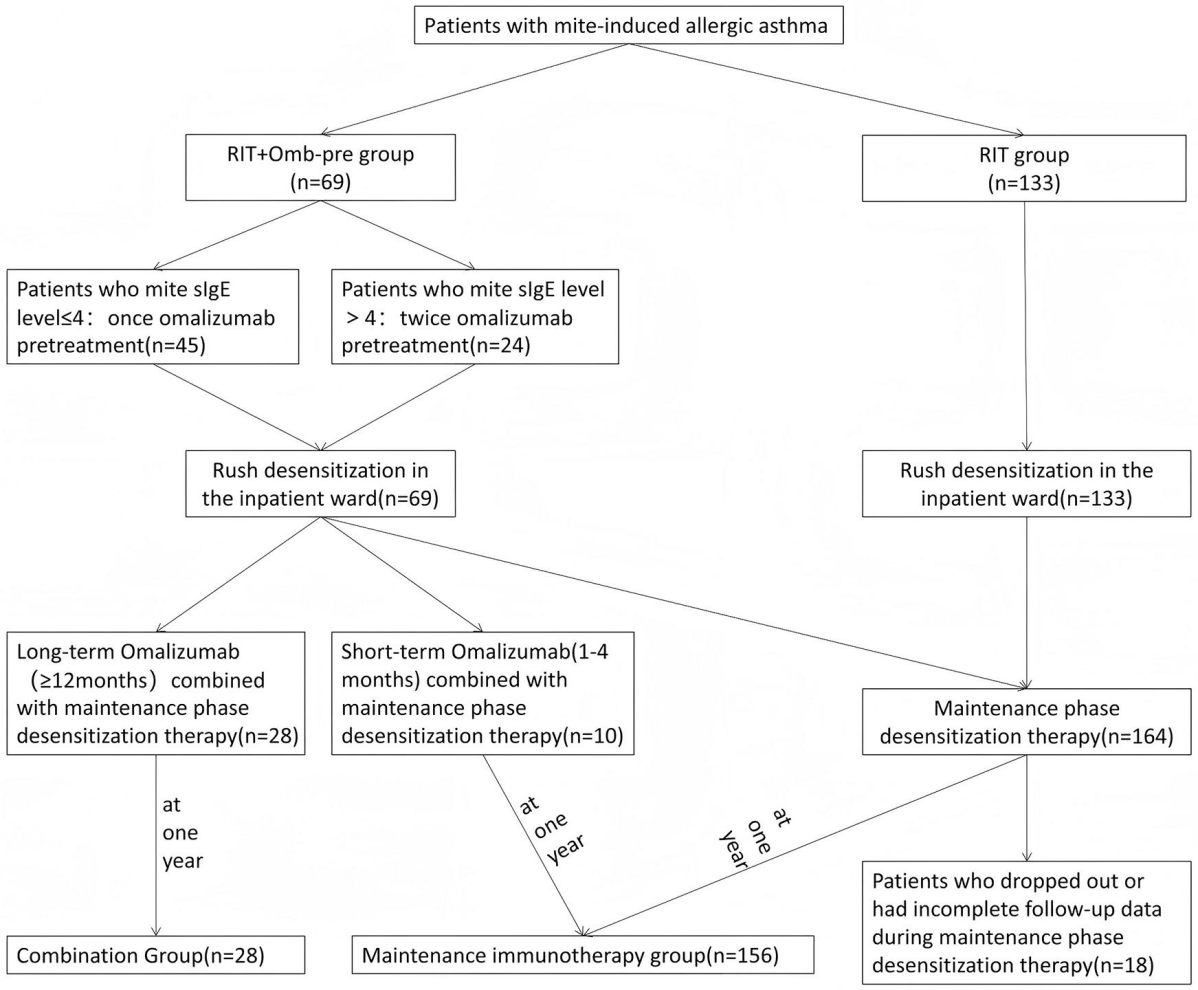
Flowchart of allergen immunotherapy. This study included 202 patients (aged 5–60 years) with mite-induced allergic asthma between January 2018 and January 2024. Patients were assigned to 2 treatment groups based on the treatment regimen they received: the RIT monotherapy group received RIT alone, and RIT + Omb-pre group pretreated with omalizumab before RIT. After completion of the rush immunotherapy phase, patients were followed for 12 months to assess clinical efficacy outcomes. Of the 184 patients who completed 1-year maintenance therapy post-RIT, 28 received 12-month omalizumab with desensitization (Combination group), while 10 underwent short-term omalizumab (1–4 months) prior to continuing desensitization alone. Given omalizumab's transient efficacy, the short-term (*n* = 10) and desensitization-only (*n* = 146) groups were combined as the Maintenance Immunotherapy group. Eighteen patients were excluded due to treatment discontinuation or incomplete follow-up data. RIT, rush immunotherapy.

This is a single-center, real world, retrospective study performed at the Henan Provincial People's Hospital Affiliated to Zhengzhou University, Henan, China. This study included patients (aged 5–60 years) with mite-induced allergic asthma who received treatment at our hospital between January 2018 and January 2024. The treatment consisted of either RIT combined with omalizumab pretreatment (RIT + Omb-pre), or RIT alone (Figure 1). The diagnosis of asthma was initially confirmed according to the Global Initiative for Asthma (GINA) criteria (13), with disease severity being periodically reassessed using the GINA assessment matrix for all patients. Patients with severe asthma were excluded. All patients underwent standardized allergen testing (skin prick tests and serum sIgE assays) to establish either exclusive mite sensitization, or mite dominance with co-sensitization to 2–3 other allergens based on clinical relevance. Mite allergy confirmation required: (1) skin prick test positivity (≥++), and (2) mite-specific IgE level (ImmunoCAP level ≥1). Serum allergen-specific IgE levels were quantified using the ImmunoCAP System (Thermo Fisher Scientific, Sweden).

Patients were assigned to 2 treatment groups based on the treatment regimen they received: the RIT monotherapy group received RIT alone, and RIT + Omb-pre group pretreated with omalizumab before RIT. In the RIT + Omb-pre group, the initiation timeline for RIT was stratified based on mite-specific IgE (sIgE) levels as follows: (1) the sIgE level ≤4: RIT was initiated 14 days after once administration of omalizumab, (2) the sIgE level 5–6: Following two monthly administrations of omalizumab, RIT was started 14 days later (Figure 1). Omalizumab dosing followed the standard asthma protocol, with the dosage determined according to guidelines based on body weight and total IgE levels (14).

This study protocol was reviewed and approved by the Institutional Review Board of The Henan Provincial People's Hospital Affiliated to Zhengzhou University, in full compliance with the Declaration of Helsinki and China's Regulations on Human Genetic Resources. Written informed consent encompassing both treatment authorization (pre-baseline) and separate data/publication permissions was obtained from all participants. Written informed consent was obtained from legal guardians for all enrolled minors before study initiation.

### Treatment

To ensure safety during RIT, all patients received standardized premedication with oral H1-antihistamines each morning 60 min prior to allergen extract administration. Post-injection monitoring protocols mandated continuous observation for 60 min, with systematic assessment of local and systemic reactions at 15-min intervals. The RIT protocol was completed over six consecutive days, with a total of 14 injections scheduled: 3 injections daily on the first three days, 2 injections daily on the fourth and fifth days, and 1 injection on the sixth day, with intervals of at least 2 h between each injection. All rush desensitization treatments were administered in the inpatient clinical ward with continuous cardiorespiratory monitoring.

The AIT in this study utilized standardized mite extracts containing a 50%:50% volume-to-volume mixture of Dermatophagoides pteronyssinus (Der p) and Dermatophagoides farinae (Der f) (Allergopharma®, Germany), administered subcutaneously according to EAACI immunotherapy guidelines (15). The highest concentration of the mite allergen shot used had an allergenic activity of 5,000 TU/ml. The first maintenance dose was administered on day 6 for patients in both treatment arms. Following the initial maintenance dose, patients transitioned to the sustained maintenance phase of immunotherapy, receiving standardized subcutaneous injections of 1.0 ml allergen extract (5,000 TU/ml) administered every 4 weeks. For patients who failed to achieve the target allergen peak concentration during the rush desensitization phase, maintenance therapy was initiated at their individual maximum tolerated dose. Gradual dose escalation was subsequently implemented during the maintenance phase. All maintenance-phase desensitization therapies were conducted in outpatient settings. The detailed RIT schedule were described in Table 1.

**Table 1:** Rush immunotherapy schedule.

Time	Injection no.	Number of vials	Volume per vial (ml)
Day 1	1	1	0.1
	2	2	0.1
	3	2	0.2
Day 2	4	2	0.4
	5	2	0.6
	6	2	0.8
Day 3	7	3	0.1
	8	3	0.2
	9	3	0.3
Day 4	10	3	0.4
	11	3	0.5
Day 5	12	3	0.5
	13	3	0.5
Day 6	14	3	1.0

### Assessment of safety and efficacy

The safety during RIT was comprehensively assessed by quantifying the incidence of systemic adverse reaction (SR), with specific SR types retrospectively categorized through structured extraction from electronic health records; the severity of SR linked to RIT was rigorously classified according to the World Allergy Organization (WAO) criteria (16). Emergency protocols were activated immediately upon SR occurrence. The completion status of the expected peak concentration in RIT has also been thoroughly documented.

Pre-intervention baseline metrics—including Asthma Control Questionnaire-5 (ACQ-5) scores and daily medication scores (17) were recorded for all patients prior to RIT. Upon completion of RIT, all patients in the RIT monotherapy cohort and majority of the RIT + Omb-pre cohort initiated standardized maintenance-phase desensitization therapy at 4 week interval, while the remaining RIT + Omb-pre patients adopted two adjunctive strategies: (1) short-term omalizumab co-therapy (1–4months) followed by omalizumab discontinuation or (2) long-term omalizumab-maintenance immunotherapy synchronization (≥12 months). At 1-year follow-up, all metrics were re-assessed to determine clinical efficacy (Figure 1).

### Statistical analysis

In this study, data processing was performed using SPSS version 26.0. Categorical data were expressed as number (*n*) and percentage (%), and chi-square test was used to compare categorical variables between the 2 treatment groups. Fisher's exact test was used for categorical variables that did not meet the condition of chi-square test. Continuous data were presented as mean ± standard deviation (x ± s). For normally distributed variables with homogeneity of variance, intergroup comparisons were conducted using the independent samples *t*-test, while intragroup comparisons were performed with the paired *t*-test. Wilcoxon test was used to compare inter-group continuous variables that did not meet the conditions for *t*-test. A *P*-value <0.05 was considered statistically significant.

## Results

### Patient demographics and baseline characteristics

This study enrolled a total of 202 asthma patients underwent RIT, including 133 patients received RIT alone (RIT group) and 69 patients pretreated with omalizumab before RIT (RIT + Omb-pre group). The RIT group comprised 72 males and 61 females with a mean age of 25.51 ± 14.18 years, including 97 patients exclusively allergic to dust mites and 36 patients sensitized to additional allergens; clinical diagnoses included asthma alone (14 patients), allergic rhinitis with asthma (98 patients), allergic rhinitis with asthma and urticaria (19 patients), and allergic rhinitis with asthma with atopic dermatitis (2 patients). The RIT + Omb-pre group included 24 males and 45 females with a mean age of 25.77 ± 15.74 years, consisting of 34 patients with exclusive dust mite allergy and 35 patients sensitized to other allergens; clinical diagnoses were asthma alone (5 patients), allergic rhinitis with asthma (44 patients), allergic rhinitis with asthma and urticaria (14 patients), and allergic rhinitis with asthma with atopic dermatitis (6 patients). Both study groups were stratified into subgroups based on patient age, mite sIgE levels, the ratio of combined Dermatophagoides pteronyssinus (d1) and Dermatophagoides farinae (d2) specific IgE to total IgE(sIgE/T-IgE), and total IgE(T-IgE) levels. Patient demographics and baseline clinical characteristics were described in Table 2.

**Table 2:** Patient demographics and baseline characteristics.

Variables	RIT Group (*n* = 133)	RIT + Omb-pre Group (*n* = 69)	*p*-value
Gender (Male/Female), *n*/*n*	72/61	24/45	0.009
Mean age (year)	25.51 ± 14.18	25.77 ± 15.74	0.907
Age stratification			
5–12 years old, *n* (%)	33 (24.81%)	20 (28.99%)	0.773
13–18 years old, *n* (%)	30 (22.56%)	16 (23.19%)	
19–60 years old, *n* (%)	70 (52.63%)	33 (47.82%)	
(d1 + d2)sIgE/T-IgE			
<10%, *n* (%)	59 (44.36%)	15 (21.74%)	0.007
10%–30%, *n* (%)	42 (31.58%)	30 (43.48%)	
>30%, *n* (%)	32 (24.06%)	24 (34.78%)	
mite-specific IgE level			
Level 1–2, *n* (%)	31 (23.31%)	5 (7.25%)	0.013
Level 3–4, *n* (%)	70 (52.63%)	40 (57.97%)	
Level 5–6, *n* (%)	32 (24.06%)	24 (34.78%)	
T-IgE level			
<200, *n* (%)	53 (39.85%)	19 (27.53%)	0.081
200–500, *n* (%)	38 (28.57%)	30 (43.48%)	
>500, *n* (%)	42 (31.58%)	20 (28.99%)	
Multiple sensitization			
Yes, *n* (%)	36 (27.07%)	35 (50.72%)	0.001
No, *n* (%)	97 (72.93%)	34 (49.28%)	
Diagnosis			
AA	14 (10.53%)	5 (7.24%)	0.044
AA + AR	98 (73.68%)	44 (63.77%)	
AA + AR + UT	19 (14.29%)	14 (20.29%)	
AA + AR + AD	2 (1.50%)	6 (8.70%)	

Continuous variables were expressed as means ± SD, while categorical variables were expressed as N (%).

RIT, rush immunotherapy; Omb, omalizumab. RIT group received RIT alone, and RIT + Omb-pre group received RIT combined with omalizumab pretreatment.

d1, dermatophagoides pteronyssinus; d2, dermatophagoides farinae.

mite-specific IgE level, the higher sIgE level between d1 and d2 is selected.

AR, allergic rhinitis; AA, allergic asthma; UT, urticaria; AD, atopic dermatitis.

### Safety of RIT

During RIT, most patients experienced localized adverse reactions of varying severity. This study focused on evaluating the risk of RIT through systemic adverse reactions (SRs). In the RIT group (133 patients), 1,826 allergen injections were administered during the rush desensitization protocol. 61 SRs events (3.34% of the injections) were observed in 33 (33/133, 24.81%) patients, including 53 Grade 1 reactions and 8 Grade 2 reactions, with no reactions exceeding Grade 2. A total of 113 patients (113/133, 84.96%) successfully completed the target peak concentration of rush desensitization. In the RIT + Omb-pre group (69 patients), 954 allergen injections were administered, with 12 SRs events(1.26% of the injections) were observed in 8 patients (8/69, 11.59%), including 10 Grade 1 reactions and 2 Grade 2 reactions, and no reactions above Grade 2. 65 patients(65/69, 94.20%) in this group successfully achieved the target peak concentration. Compared to the RIT group, the RIT + Omb-pre regimen significantly lowered the incidence of SRs and yielded a higher completion rate of the target peak concentration (*p* < 0.05 and *p* = 0.054, respectively; Table 3).

**Table 3:** Systemic adverse reactions in the RIT and RIT + Omb-pre groups.

Adverse reactions	RIT group (*n* = 133)		RIT + Omb-pre group (*n* = 69)	
	SR-events	SR-patients	SR-events	SR-patients
SRs	61 (3.34%)^a^	33 (24.81%)^a^	12 (1.26%)	8 (11.59%)
Grade 1	53 (2.90%)	33 (24.81%)	10 (1.05%)	8 (11.59%)
Grade 2	8 (0.44%)	8 (6.02%)	2 (0.21%)	2 (2.90%)
Grade 3	0	0	0	0
Grade 4	0	0	0	0

SRs, systemic adverse reactions.

SR-Events, systemic adverse reaction events during rush immunotherapy, *n* (% of total injections).

SR-Patients, patients who developed systemic adverse reactions during rush immunotherapy, *n* (%).

a Significant difference compared to RIT + Omb-pre group.

### Association of RIT safety with mite sIgE levels, sIgE/T-IgE ratio, and T-IgE

This study stratified patients from both the RIT group and the RIT + Omb-pre cohort into distinct subgroups based on mite sIgE levels, sIgE/T-IgE ratio, and T-IgE to delineate their collective associations with SR risks during RIT.

In evaluating the association between mite sIgE levels and SR risks during RIT, the RIT group exhibited significant differences across sIgE subgroups. SR events occurred in 2 (0.46%of injections), 27 (2.80% of injections), and 32 (7.49% of injections) injections in the sIgE 1–2, 3–4, and 5–6 level subgroups, respectively (*p* < 0.05 for all intergroup comparisons). Similarly, the proportion of patients experienced SRs varied markedly among subgroups: 1 (3.23%), 15 (21.43%), and 17 (53.13%) in the sIgE 1–2, 3–4, and 5–6 level subgroups (*p* < 0.05). The completion rate of peak immunotherapy concentration was significantly higher in the sIgE 1–2 and 3–4 level subgroups than in the 5–6 level subgroup (*p* < 0.05). In contrast, the RIT + Omb-pre group showed no correlation between sIgE stratification and SR events frequency, percentage of SR-affected patients, or peak concentration completion rates. Comparative analysis revealed that, relative to the RIT group, the RIT + Omb-pre group achieved significant reductions in SR events frequency (1.20% vs. 7.49%; *p* < 0.05) and percentage of SR-affected patients (12.50% vs. 53.13%; *p* < 0.05), alongside a markedly improved peak concentration completion rate (91.67% vs. 65.63%; *p* < 0.05) in the sIgE 5–6 level subgroup. For the sIgE 3–4 level subgroup, SR events frequency decreased marginally (1.45%vs. 2.80%; *p* = 0.09), with non-significant numerical improvements in percentage of SR-affected patients (12.50% vs. 21.43%; *p* > 0.05) and peak concentration completion rates (95.00% vs.87.14%; *p* > 0.05). No significant differences were observed in the sIgE 1–2 level subgroup for SR events frequency (0.00%vs. 0.46%; *p* > 0.05), percentage of SR-affected patients (0.00% vs. 3.23%; *p* > 0.05), or peak concentration completion rate (100% vs. 100%; *p* > 0.05) (Table 4).

**Table 4:** Risk stratification in the RIT and RIT + Omb-pre groups.

Subgroup	RIT group (*n* = 133)			RIT + Omb-pre group (*n* = 69)		
	SR-events	SR-patients	Peak	SR-events	SR-patients	Peak
Mite sIgE level						
subset(1): Level 1–2	2 (0.46%)^a^^,^^b^^,^^f^	1 (3.23%)^a^^,^^b^^,^^f^	31 (100%)^d^^,^^b^^,^^f^	0 (0.00%)^d^^,^^e^	0 (0.00%)^d^^,^^e^	5 (100%)^d^^,^^e^
subset(2): Level 3–4	27 (2.80%)^b^^,^^f^	15 (21.43%)^b^^,^^f^	61 (87.14%)^b^^,^^f^	8 (1.45%)^e^	5 (12.50%)^e^	38 (95.00%)^e^
subset(3): Level 5–6	32 (7.49%)^c^	17 (53.13%)^c^	21 (65.63%)^c^	4 (1.20%)	3 (12.50%)	22 (91.67%)
(d1 + d2)sIgE/T-IgE						
subset(1): <10%	9 (1.10%)^a^^,^^b^^,^^f^	5 (8.47%)^a^^,^^b^^,^^f^	56 (94.92%)^d^^,^^b^^,^^f^	4 (1.96%)^d^^,^^e^	2 (13.33%)^d^^,^^e^	13 (86.67%)^d^^,^^e^
subset(2): 10%–30%	19 (3.28%)^b^^,^^c^	11 (26.19%)^b^^,^^c^	35 (83.33%)^e^^,^^f^	4 (0.96%)^e^	2 (6.67%)^e^	29 (96.67%)^e^
subset(3): >30%	33 (7.75%)^c^	17 (53.13%)^c^	22 (68.75%)^c^	4 (1.20%)	4 (16.67%)	23 (95.83%)
T-IgE level						
subset(1): <200	20 (2.72%)^d^^,^^e^^,^^f^	11 (20.75%)^d^^,^^e^^,^^f^	49 (92.45%)^d^^,^^b^^,^^f^	4 (1.50%)^d^^,^^e^	3 (15.79%)^d^^,^^e^	19 (100%)^d^^,^^e^
subset(2): 200–500	16 (3.05%)^e^^,^^f^	8 (21.05%)^e^^,^^f^	32 (84.21%)^e^^,^^f^	5 (1.22%)^e^	3 (10.00%)^e^	27 (90.00%)^e^
subset(3): >500	25 (4.40%)^c^	14 (33.33%)^c^	32 (76.19%)^f^	3 (1.08%)	2 (10.00%)	19 (95.00%)

d1, dermatophagoides pteronyssinus; d2, dermatophagoides farinae.

Mite sIgE level, the higher sIgE level between d1 and d2 is selected.

SR-Events, systemic adverse reactions events during rush immunotherapy, *n* (% of total injections).

SR-Patients, patients who developed systemic adverse reactions during rush immunotherapy, *n* (%).

Peak, patients who successfully achieved the target peak concentration, *n* (%).

a Significant difference compared to subset (2) within the same subgroup.

b Significant difference compared to subset (3) within the same subgroup.

c Significant difference compared to RIT + Omb-pre group.

d No significant difference compared to subset (2) within the same subgroup.

e No significant difference compared to subset (3) within the same subgroup.

f No significant difference compared to RIT + Omb-pre group.

In assessing the association between (d1 + d2) sIgE/T-IgE ratio and SR risks during RIT, the RIT group exhibited significant differences across ratio-based subgroups. SR events occurred in 9 (1.10% of injections), 19 (3.28% of injections), and 33 (7.75% of injections) injections in the <10%, 10%–30%, and >30% ratio subgroups, respectively (*p* < 0.05 for all intergroup comparisons). Similarly, the proportion of patients experienced SRs differed markedly: 5 (8.47%), 11 (26.19%), and 17 (53.13%) in the <10%, 10%–30%, and >30% ratio subgroups (*p* < 0.05). The completion rate of peak desensitization concentration was significantly higher in the <10% ratio subgroup vs. the >30% ratio subgroup (*p* < 0.05). In contrast, the RIT + Omb-pre group showed no correlation between sIgE/T-IgE ratio stratification and SR events frequency, SR-affected patients proportion, or peak concentration completion rates. Comparative analysis revealed that, relative to the RIT group, the RIT + Omb-pre group achieved significant reductions in SR events frequency (1.20% vs. 7.75%; *p* < 0.05) and SR-affected patient proportion (16.67% vs. 53.13%; *p* < 0.05), alongside a markedly improved peak desensitization completion rate (95.83% vs. 68.75%; *p* < 0.05) in the >30% ratio subgroup. For the 10%–30% ratio subgroup, SR events frequency (0.96% vs. 3.28%, *p* < 0.05) and SR-affected patient proportion(6.67% vs. 26.19%; *p* < 0.05) decreased significantly, with non-significant numerical improvements in peak completion rates (96.67% vs. 83.33%; *p* > 0.05). No significant differences were observed in the <10% ratio subgroup for SR events frequency (1.96% vs. 1.10%; *p* > 0.05), SR-affected patient proportion (13.33% vs. 8.47%; *p* > 0.05), or peak completion rates (86.67% vs. 94.92%; *p* > 0.05) (Table 4).

In the stratified analysis of T-IgE subgroups, neither the RIT group nor the RIT + Omb-pre group exhibited statistically significant differences in SR rates or peak desensitization concentration completion rates across subgroups. However, comparative analysis between the two groups revealed that in patients with high T-IgE levels (T-IgE>500 ku/ml), the RIT + Omb-pre group demonstrated a statistically significant reduction in both the incidence of SR events (1.08% vs. 4.40%, *p* < 0.05) and the proportion of patients experienced SRs (10.00% vs. 33.33%, *p* < 0.05) compared to the RIT group (Table 4).

### Association between safety of RIT and age stratification

To assess age-related SR risks during RIT, patients in RIT group were stratified into children (5–12 years), teenagers (13–18 years), and adults (19–60 years). Intragroup comparisons showed no significant differences between children and teenagers in SR events frequency, SR-affected patients proportion, or peak completion rates (all *p* > 0.05). Both pediatric subgroups (children/teenagers) exhibited significantly higher SR incidence (*p* < 0.05) and SR patient proportions (*p* < 0.05) than adults. The peak concentration completion rate was numerically lower in the children subgroup than in adults (*p* > 0.05), while teenagers had a significantly lower rate compared to adults (*p* < 0.05) (Table 5).

**Table 5:** Age stratification in the RIT and RIT + Omb-pre groups.

Subgroup	RIT group (*n* = 133)			RIT + Omb-pre group (*n* = 69)		
	SR-events	SR-patients	Peak	SR-events	SR-patients	Peak
Children (5–12years)						
Mite sIgE level: 1–2	0 (0.00%)^c^^,^^d^	0 (0.00%)^c^^,^^d^	5 (100%)^c^^,^^d^	–	–	–
Mite sIgE level:3–6	21 (5.50%)^a^^,^^b^^,^^c^	12 (42.86%)^a^^,^^b^^,^^c^	22 (78.57%)^c^^,^^d^^,^^e^	3 (1.09%)^c^^,^^d^	2 (10.00%)^c^^,^^d^	19 (95.00%)^c^^,^^d^
Subtotal	21 (4.65%)^a^^,^^b^^,^^c^	12 (36.36%)^a^^,^^b^^,^^c^	27 (81.81%)^c^^,^^d^^,^^e^	3 (1.09%)^c^^,^^d^	2 (10.00%)^c^^,^^d^	19 (95.00%)^c^^,^^d^
Teenagers (13–18years)						
Mite sIgE level: 1–2	0 (0.00%)^d^	0 (0.00%)^d^	5 (100%)^d^	–	–	–
Mite sIgE level:3–6	20 (5.95%)^a^^,^^b^	11 (44.00%)^a^^,^^e^	17 (68.00%)^d^^,^^e^	4 (1.79%)^d^	3 (18.75%)^d^	15 (93.75%)^d^
Subtotal	20 (4.93%)^a^^,^^b^	11 (36.67%)^a^^,^^e^	22 (73.33%)^a^^,^^e^	4 (1.79%)^d^	3 (18.75%)^d^	15 (93.75%)^d^
Adults (19–60years)						
Mite sIgE level: 1–2	2 (0.68%)^e^	1 (4.76%)^e^	21 (100%)^e^	0 (0.00%)	0 (0.00%)	5 (100%)
Mite sIgE level:3–6	18 (2.67%)^e^	9 (18.37%)^e^	43 (87.76%)^e^	5 (1.30%)	3 (10.71%)	26 (92.86%)
Subtotal	20 (2.07%)^e^	10 (14.29%)^e^	64 (91.42%)^e^	5 (1.10%)	3 (9.09%)	31 (93.93%)

Mite sIgE level, the higher sIgE level between Dermatophagoides pteronyssinus and Dermatophagoides farinae is selected.

SR-events, systemic adverse reactions events during rush immunotherapy, *n* (% of total injections).

SR-patients, patients who developed systemic adverse reactions during rush immunotherapy, *n* (%).

Peak, patients who successfully achieved the target peak concentration, *n* (%).

a Significant difference compared to the Adults(19–60years) subgroup.

b Significant difference compared to RIT + Omb-pre group.

c No significant difference compared to the Teenagers (13–18years) subgroup.

d No significant difference compared to the Adults (19–60years) subgroup.

e No significant difference compared to RIT + Omb-pre group.

Age subgroup analysis by mite sIgE severity: In the mite sIgE level 1–2 group, no significant age-related differences were observed in SR incidence, SR patient proportions, or peak completion rates (all *p* > 0.05). For mite sIgE level 3–6 group, children and teenagers had comparable outcomes (*p* > 0.05) but higher SR incidence (*p* < 0.05) and SR patient proportions (*p* < 0.05) than adults. Their peak completion rates were numerically lower than adults’ but lacked statistical significance (*p* > 0.05) (Table 5).

Compared to RIT group, RIT + Omb-pre group significantly reduced SR incidence in pediatric patients (children/teenagers; *p* < 0.05). In adults, RIT + Omb-pre group showed only a marginal SR reduction (*p* > 0.05) (Table 5).

### Association between AIT outcomes and age as well as sIgE/T-IgE ratio

Of the 184 patients who completed 1-year maintenance therapy post-RIT, 28 received 12-month omalizumab with desensitization (Combination group), while 10 underwent short-term omalizumab (1–4 months) prior to continuing desensitization alone. Given omalizumab's transient efficacy, the short-term (*n* = 10) and desensitization-only (*n* = 146) groups were combined as the Maintenance Immunotherapy group. Eighteen patients were excluded due to treatment discontinuation or incomplete follow-up data (Figure 1).

To assess the age-related efficacy of desensitization, the Maintenance Immunotherapy group was stratified into children, teenager, and adult subgroups. All subgroups and the Combination group showed significant improvements in daily medication scores and asthma ACQ-5 scores at 1 year vs. baseline, although the improvements were significantly greater in the Combination group. Within the Maintenance Immunotherapy group, children and teenager subgroups showed comparable improvements in asthma ACQ-5 scores, both significantly superior to the adult subgroup, while for daily medication scores, the children subgroup had a numerically larger reduction than the teenager and adult subgroups, though without statistical significance (Table 6).

**Table 6:** Age-stratified efficacy of desensitization therapy.

Subgroup	Daily medication score			ACQ-5 score		
	Baseline	Month 12	Change (Δ)	Baseline	Month 12	Change (Δ)
Children group (*n* = 37)	5.27 ± 1.57	2.32 ± 1.55^d^	2.95 ± 1.20	1.53 ± 0.74	0.54 ± 0.28^d^	0.99 ± 0.64
Teenager group (*n* = 30)	6.27 ± 1.36	3.50 ± 1.68^d^	2.77 ± 1.04^e^	1.60 ± 0.48	0.71 ± 0.34^d^	0.89 ± 0.36^e^
Adult group (*n* = 89)	6.53 ± 1.78	3.92 ± 1.63^d^	2.61 ± 1.18^e^^,^^f^	1.40 ± 0.47	0.77 ± 0.31^d^	0.63 ± 0.37^a^^,^^b^
Combination group (*n* = 28)	7.36 ± 1.42	1.82 ± 1.28^d^	5.54 ± 1.45^a^^,^^b^^,^^c^	1.65 ± 0.57	0.34 ± 0.22^d^	1.31 ± 0.54^a^^,^^b^^,^^c^

ChangeΔ = Month 12 – Baseline.

a Significant difference compared to the Children group.

b Significant difference compared to the Teenager group.

c Significant difference compared to the Adult group.

d Significant difference compared to Baseline.

e No significant difference compared to the Children group.

f No significant difference compared to the Teenager group.

To assess the association between desensitization efficacy and the sIgE/T-IgE ratio, patients in the Maintenance Immunotherapy group were stratified into two subgroups: (d1 + d2)sIgE/T-IgE <16% and (d1 + d2)sIgE/T-IgE >16%. Both subgroups and the Combination group demonstrated significant improvements in daily medication scores and asthma ACQ-5 scores at 1 year vs. baseline, although the improvements were significantly greater in the Combination group. Within the Maintenance group, the >16% subgroup exhibited significantly greater reductions in both ACQ-5 and daily medication scores than the <16% subgroup (Table 7).

**Table 7:** Sige/tIgE ratio-stratified efficacy of desensitization therapy.

Subgroup	Daily medication score			ACQ-5 score		
	Baseline	Month 12	Change (Δ)	Baseline	Month 12	Change (Δ)
(d1 + d2)sIgE/T-IgE<16% group (*n* = 87)	6.10 ± 1.90	3.70 ± 1.89^c^	2.40 ± 1.15	1.40 ± 0.50	0.77 ± 0.33^c^	0.63 ± 0.38
(d1 + d2)sIgE/T-IgE>16% group (*n* = 69)	6.28 ± 1.50	3.16 ± 1.47^c^	3.12 ± 1.06^a^	1.57 ± 0.60	0.63 ± 0.29^c^	0.94 ± 0.52^a^
Combination group (*n* = 28)	7.36 ± 1.42	1.82 ± 1.28^c^	5.54 ± 1.45^a^^,^^b^	1.65 ± 0.57	0.34 ± 0.22^c^	1.31 ± 0.54^a^^,^^b^

d1, dermatophagoides pteronyssinus; d2, dermatophagoides farinae.

ChangeΔ = Month 12 – Baseline.

a Significant difference compared to the sIgE(d1 + d2)/T-IgE<16% group.

b Significant difference compared to the sIgE(d1 + d2)/T-IgE>16% group.

c Significant difference compared to Baseline.

## Discussion

AIT offers multiple advantages in the management of allergic diseases, including symptom alleviation, quality-of-life improvement, reduced corticosteroid dependence, enhanced allergen tolerance, prevention of new sensitizations, and modification of the natural progression of allergic conditions (18, 19). In recent years, accumulating evidence has reinforced the critical role of AIT in treating allergic rhinitis and asthma (20–22). However, the clinical application of conventional AIT remains limited due to its prolonged duration, delayed efficacy, and frequent administration requirements (6). RIT characterized by a significantly shortened dose-escalation phase and fewer injections, accelerates the onset of therapeutic effects and improves patient compliance (8). Despite these benefits, concerns over potential risks have hindered the widespread adoption of RIT in clinical practice, particularly due to the lack of large-scale safety data—especially in pediatric populations and highly sensitized patients. Furthermore, with the increasing use of anti-IgE therapy, whether omalizumab exhibits synergistic effects with RIT remains understudied. To evaluate the safety profile of RIT in allergic asthma—particularly in children and hypersensitive individuals—and to explore potential synergistic effects between omalizumab and RIT, this retrospective study analyzed 202 cases of RIT alone or combined with omalizumab in patients with allergic asthma. Stratified analyses were conducted based on age, mite sIgE levels, sIgE/T-IgE ratios, and T-IgE levels. Outcomes included SRs, RIT completion rates, improvements in clinical parameters following omalizumab intervention, and 1-year follow-up efficacy across subgroups.

The foremost safety concern in RIT is the occurrence of SRs. In this study, SRs predominantly occurred at doses of 0.5 ml and 1.0 ml from Allergopharma Vial No. 3. Common clinical manifestations included urticaria, cutaneous edema, and asthma exacerbations, with a minority of patients reporting gastrointestinal symptoms such as nausea, vomiting, or abdominal pain. All SRs resolved rapidly following methylprednisolone or epinephrine administration, and no grade ≥3 reactions were observed. Asthmatic patients pretreated with omalizumab prior to RIT exhibited a significant reduction in SRs incidence compared to the RIT-alone cohort. This finding aligns with global clinical evidence (7, 23–25). This protective effect can be attributed to the fundamental mechanism of action of omalizumab. As a recombinant humanized monoclonal anti-IgE antibody, omalizumab neutralizes free IgE, blocking its attachment to the high-affinity receptor FcεRI on mast cells and basophils. This direct inhibition, coupled with the subsequent downregulation of FcεRI expression, stabilizes these cells and suppresses the release of downstream allergic mediators. This mechanism effectively reduces the risk of SRs during AIT and alleviates underlying allergic inflammation (11, 26, 27).

The subgroup analysis of this study further revealed that the incidence of SRs during RIT was positively correlated with mite sIgE levels and the ratio of sIgE/T-IgE, but not associated with T-IgE levels. Asthmatic patients with mite sIgE grades 1–2 and an sIgE/T-IgE ratio <10% exhibited a lower incidence of SRs during RIT and the addition of omalizumab did not further reduce these reactions. In contrast, patients with mite sIgE grades 3–4 and 5–6, as well as those with an sIgE/T-IgE ratio of 10%–30% and >30%, showed a significantly higher incidence of SRs during RIT, though concomitant use of omalizumab markedly reduced such risks. Based on these research findings, we recommend that the World Allergy Organization and the European Academy of Allergy and Clinical Immunology consider the following recommendations when developing guidelines for rush desensitization therapy: For patients with sIgE grades 1–2 and an sIgE/T-IgE ratio <10%, RIT demonstrates satisfactory safety without requiring additional omalizumab for adverse reaction prevention; for those with sIgE grades 3–4 and an sIgE/T-IgE ratio of 10%–30%, omalizumab should be considered as adjunctive therapy due to the elevated risk of systemic adverse events;for high-risk patients with sIgE grades 5–6 and an sIgE/T-IgE ratio >30%, our data suggest that intensive pretreatment with omalizumab is strongly recommended prior to initiating rush desensitization, with real-time adjustments to the desensitization protocol based on individual patient responses during treatment.

Currently, there is limited global research on the relationship between RIT risks and age, with most studies focusing exclusively on pediatric or adult populations and rarely comparing these groups within the same cohort (7, 28, 29). In this study, age-stratified analysis demonstrated that both children and teenagers had significantly higher SR rates during RIT than adults. Furthermore, omalizumab administration resulted in a more pronounced reduction in adverse reactions among children and teenager patients compared to adults. These findings suggest that adults tolerate rush desensitization better than children and teenagers, likely due to factors such as body weight, muscle mass, and immune system maturity. Based on these findings, we propose that enhanced omalizumab pretreatment should be prioritized for highly sensitized pediatric patients (relative to adults) before rush desensitization to minimize SR incidence.

Regarding predictive biomarkers for desensitization therapy efficacy, Dr. Gabriele Di Lorenzo's research team demonstrated through large-scale data that patients with a sIgE/T-IgE ratio exceeding 16.2% exhibited significantly enhanced treatment responsiveness (30). Similarly, Dr. Mengrong Li's group in China identified an sIgE/T-IgE ratio above 15.0% as the optimal predictive cutoff value for clinical effectiveness (31). Our study corroborates these findings, showing that the subgroup with sIgE/T-IgE ratios >16% achieved substantially greater improvements in ACQ scores and daily medication burden compared to those with ratios <16% during the 12-month intervention. Furthermore, this study reaffirmed the age-dependent efficacy correlation, with pediatric patients demonstrating superior therapeutic outcomes to adult patients. Notably, in the 1-year follow-up cohort receiving concurrent omalizumab therapy throughout the desensitization process, these patients exhibited the most pronounced improvements in both ACQ and medication scores among all groups, confirming the synergistic effects of combined omalizumab and desensitization therapy.

It is important to acknowledge several limitations in this study. First, the single-center retrospective design lacked rigorous double-blind, placebo-controlled methodology. The study population was restricted to asthmatic patients undergoing mite desensitization, with no comparative data from pollen-, mold-, or pet-allergy cohorts. Although the overall sample size was substantial, certain subgroup analyses were limited by small subgroup populations or imbalanced demographic distributions across subgroups. Finally, the evaluation of desensitization efficacy was constrained by a relatively short follow-up duration and a restricted range of outcome measures. Therefore, further multicenter prospective trials with larger cohorts, extended follow-up periods (e.g., ≥24 months), and comprehensive biomarker assessments are warranted to systematically evaluate both the risks of rush desensitization and the clinical benefits of combining omalizumab with this therapy.

As a retrospective real-world investigation, this study is inherently subject to certain clinical biases. The assignment to the RIT + Omb-pre group or the RIT group was not completly randomized but was based on the treating physician's comprehensive clinical assessment at the time. Typically, patients with more severe asthma symptoms, higher baseline allergen-specific IgE levels, higher sIgE/T-IgE ratio, or those deemed at higher risk for RIT-related SRs were more likely to be prescribed the combination therapy with omalizumab pretreatment. This clinical decision-making process explains the significant differences in baseline characteristics between the two groups. In this clinical study, compared to the RIT group, the RIT + Omb-pre group contained a higher proportion of patients at elevated risk for RIT-related SRs. Nevertheless, the results demonstrated a lower incidence of these SRs in the RIT + Omb-pre group, which further substantiates the superiority of the RIT + Omb-pre treatment regimen. Furthermore, in the Results section, to mitigate the potential bias introduced by the baseline differences in factors such as allergen-specific IgE levels and the sIgE/T-IgE ratio between the two groups, a subgroup analysis was performed. This analysis compared the incidence of SRs among patients with different sIgE levels and sIgE/T-IgE ratios, and yielded consistent conclusions. Regarding the biases in gender and sensitization patterns between the RIT group and the RIT + Omb-pre group,chi-square tests revealed that the differences in gender and sensitization patterns had little influence on the incidence of SRs. Further logistic regression analysis revealed no significant association between gender/sensitization patterns and the incidence of SRs during RIT (Supplementary Table S1). And comparative analysis showed that the impact of gender/sensitization patterns was substantially smaller than that of key indicators such as sIgE levels and the sIgE/T-IgE ratio. Therefore, we remain fully confident in the validity of the conclusions drawn from this study.

Desensitization protocols require dynamic optimization through continuous integration of emerging evidence and technological advancements to expand their clinical applicability across diverse patient populations. Evidence-based updates incorporating precision medicine frameworks and real-world outcome monitoring are essential for advancing this therapeutic modality. This study identified risk stratification for rush desensitization across populations with varying age, sIgE levels, and sIgE/T-IgE ratios, confirming omalizumab's efficacy in mitigating adverse reactions. We recommend pretreatment with omalizumab for high-risk populations prior to initiating rush desensitization. These findings position the combination of rush desensitization and biologics as a promising new therapeutic paradigm for respiratory allergic diseases.

## Data Availability

The raw data supporting the conclusions of this article will be made available by the authors, without undue reservation.
