# Supplementary material for: Real-world retrospective study on the efficacy and safety of anti-IgE therapy combined with rush immunotherapy in allergic asthma
Source: Front Allergy. 2025 Dec 15;6:1717446. doi: 10.3389/falgy.2025.1717446 (PMC12745253; doi:10.3389/falgy.2025.1717446)
Supplement: Supplementary file 1 [file Table1.docx]

| Table S1 Logistic Regression Analysis | | | | | | | |
| --- | --- | --- | --- | --- | --- | --- | --- |
|  | β | SE | z  | Wald χ2  | p | OR | OR 95% CI |
| gender | -0.233 | 0.395 | -0.589 | 0.347 | 0.556 | 0.793 | 0.366 ~ 1.718 |
| multiple sensitization | -0.066 | 0.415 | -0.16 | 0.026 | 0.873 | 0.936 | 0.415 ~ 2.109 |
| AIT Protocol | -1.467 | 0.475 | -3.09 | 9.55 | 0.002 | 0.231 | 0.091 ~ 0.585 |
| mite-specific IgE level | 0.57 | 0.178 | 3.205 | 10.269 | 0.001 | 1.768 | 1.248 ~ 2.505 |
| (d1+d2)sIgE/T-IgE | 2.62 | 1.167 | 2.245 | 5.038 | 0.025 | 13.73 | 1.394 ~ 135.239 |

Note: The dependent variable was defined as the occurrence of systemic reactions (SRs).
